# Tracing Long-Term Outcomes of Basic Research Using Citation Networks

**DOI:** 10.3389/frma.2020.00005

**Published:** 2020-09-08

**Authors:** James Onken, Andrew C. Miklos, Richard Aragon

**Affiliations:** ^1^Research Enterprise Analytics, LLC, Rockville, MD, United States; ^2^Division of Data Integration, Modeling, and Analytics, National Institute of General Medical Sciences, Bethesda, MD, United States

**Keywords:** science of science, citation networks, knowledge diffusion, research evaluation, research outcomes, basic research, government funding

## Abstract

In recent years, the science of science policy has been facilitated by the greater availability of and access to digital data associated with the science, technology, and innovation enterprise. Historically, most of the studies from which such data are derived have been econometric or “scientometric” in nature, focusing on the development of quantitative data, models, and metrics of the scientific process as well as outputs and outcomes. Broader definitions of research impact, however, necessitate the use of qualitative case-study methods. For many years, U.S. federal science agencies such as the National Institutes of Health have demonstrated the impact of the research they support through tracing studies that document critical events in the development of successful technologies. A significant disadvantage and barrier of such studies is the labor-intensive nature of a case study approach. Currently, however, the same data infrastructures that have been developed to support scientometrics may also facilitate historical tracing studies. In this paper, we describe one approach we used to discover long-term, downstream outcomes of research supported in the late 1970's and early 1980's by the National Institute of General Medical Sciences, a component of the National Institutes of Health.

## Introduction

For more than a decade, beginning when Dr. Jack H. Marburger III, the President's Science Advisor and Director of the Office of Science and Technology Policy called for a “science of science (SoS) policy” (Office of Science Technology Policy, 2006), there has been a growing community of practice in the US surrounding the evaluation of scientific research programs. Interest in SoS was both reflected in, and further stimulated by, creation of the Science of Science Innovation and Policy (SciSIP) program at the National Science Foundation (NSF) in 2006 (National Research Council, 2014a). Through its grant program, SciSIP fostered the development of data, tools, and methods “to inform the nation's public and private sectors about the processes through which investments in science and engineering (S&E) research are transformed into social and economic outcomes” (National Science Foundation, 2007). The importance of these activities is further strengthened by the involvement of other federal agencies in SciSIP, such as the National Institute of General Medical Sciences (NIGMS), a component of the National Institutes of Health (NIH) (National Institute of General Medical Sciences, 2019a). Interest in SoS—also sometimes referred to as “research on research” or “meta-research” (Kamenetzky and Hinrichs-Krapels, 2020)—has not been limited to the US. Twelve countries and regions from around the world are partners in the Research on Research Institute, established in 2019 by the Wellcome Trust, Digital Science, and the Universities of Sheffield and Leiden (Skelton, 2019).

The increase in SoS studies has been fueled, in part, by greater access to digital data on the science, technology, and innovation enterprise (National Research Council, 2014b; Fortunato et al., 2018; Waldman and Lariviere, 2020). As more sophisticated databases, tools, and methods have become available, expectations—and sometimes requirements—for public science funding agencies to document the outcomes of national investments in research have increased (Husbands Fealing et al., 2011; Oancea, 2013; Kamenetzky and Hinrichs-Krapels, 2020).

Some agencies have responded by strengthening their own data infrastructure to facilitate SoS studies. In the UK, routine collection of research impact data has expanded through the use of national databases such as researchfish® (Raftery et al., 2016). In the US, the NIH has been leading the effort of several science agencies to construct the Federal Research Portfolio Online Reporting Tools: Expenditures and Results (RePORTER) website, a database of federal research investments and associated outputs (scientific publications) (National Institutes of Health, 2019). Federal RePORTER was modeled on NIH's own RePORTER system, which links NIH-funded projects to resulting publications and patents (National Institutes of Health, 2020a). Also, the NIH Office of Portfolio Analysis has created both internal and publicly available portfolio analysis tools and data, such as the NIH Open Citation Collection (Hutchins et al., 2019) and iCite, a query and analysis tool (National Institutes of Health, 2020b). The NIH Office of Extramural Research also has created an internal NIH Portfolio Analysis and Reporting Data Infrastructure (PARDI) that combines grant records, NIH-supported publications and patents, and citation data for use by NIH staff (Zuckerman et al., 2015).

Historically, SciSIP has been largely focused on econometric or “scientometric” research: the development of quantitative data, models, and metrics of the scientific process, outputs, and outcomes (National Academies of Sciences, 2017). There have been long-standing concerns surrounding the interpretation and use of some metrics (Donovan, 2007), and a rise in their application coincided with the creation in 2015 of the Leiden Manifesto, a set of principles to guide the use of metrics so that “researchers can hold evaluators to account, and evaluators can hold their indicators to account” (Hicks et al., 2015). Despite the SoS community's increased focus on metrics, the first principle in the Manifesto emphasizes the primacy of qualitative assessment, which quantification can support but not replace.

Broader definitions of research “impact” beyond economic measures to include social, cultural, and environmental returns have also necessitated the use of qualitative case-study methods (Kearnes and Wienroth, 2011), such as the Payback Framework, which has been used in several countries to assess the impact of health-related research (Buxton and Hanney, 1996; Donovan, 2011; Donovan and Hanney, 2011). Case studies formed the basis for the UK's Research Excellence Framework beginning in 2014 (King's College London Digital Science, 2015; Research Excellence Framework, 2015). That same year in the US, the NIH Scientific Management Review Board, charged with reviewing approaches to assess the value of biomedical research, concluded that “[n]arratives constructed from well-designed case studies can be especially effective illustrations of the broad impacts of biomedical research” (National Institutes of Health, 2014). In a similar vein, NSF recently changed the name and focus of the SciSIP program to “Science of Science: Discovery, Communication, and Impact,” which may signal less emphasis being placed on metrics and an increase in the program's focus on how to enhance the value of scientific research to the public and stakeholders (National Science Foundation, 2019).

Case studies have long been used by public science funding agencies to demonstrate the impact of the research they support. One approach commonly used is the “historical tracing” or “historiographic” method (Ruegg and Jordan, 2007), a narrative account of the value of research in creating downstream inventions, products, or social benefits by tracing a series of incremental scientific advances ending in some outcome of value, such as improved public health. Tracing studies have a long history. In the late 1960's, the US NSF supported the TRACES (Technology in Retrospect and Critical Events in Science) study, which illustrated the role of basic research in five significant technologies, including the video tape recorder, oral contraceptives, and the electron microscope (Narin, 2013). The TRACES study was a response to “Project Hindsight” a similar study conducted by the US Department of Defense to assess the impact of its basic research (Sherwin and Isenson, 1967). More recent examples of tracing studies include those produced in the US by the Centers for Disease Control and Prevention (Centers for Disease Control Prevention, 2017) and the NIH (National Institutes of Health, 2018a).

A significant disadvantage of tracing studies, and a barrier to their use among science agencies, is the labor-intensive nature of the method (Comroe and Dripps, 1976; Smith, 1987; Contopoulos-Ioannidis et al., 2003; Mayernik et al., 2016). Expert knowledge is critical in identifying significant events in the path from basic research to societal outcomes (Narin, 2013; Centers for Disease Control Prevention, 2017). Such expertise can be costly, whether it is in terms of federal staff time or the cost of hiring expert consultants. However, the same data infrastructures that have been developed to support scientometrics may also facilitate historical tracing studies. The manual search for, and documentation of, evidence that basic research has contributed to a significant scientific or technological advance might be facilitated by a data infrastructure consisting of linked databases having records of research grants, scientific publications, patents, and other artifacts captured throughout the research and development process.

A data infrastructure such as that described above might also help meet an even greater challenge: continuously monitoring downstream technological advances to understand whether or to what extent they might have drawn on the results of a specific portfolio of basic research. Many historical tracing studies begin with a significant advance and trace backwards to identify the research on which it was based. For example, to demonstrate the impact of its research, NIH began with the development of childhood Haemophilus influenzae type b vaccines and worked backwards to identify prior vaccine development and the foundational research supported by NIH (National Institutes of Health, 2018b). Even when linked data sources are used, the tracing process has typically begun with the endpoint and worked backwards [see for example, Williams et al. (2015); Keserci et al. (2017)].

In contrast, forward tracing is a process of discovery beginning with a well-defined set of inputs whose outcomes have yet to be identified [see, for example, Wooding et al. (2011)]. A portfolio of research, embodied in a group of research grants or journal articles, can be traced through multiple generations of references to that work in subsequent journal articles, patents, clinical trials, clinical practice guidelines, drug products, etc.

One challenge in forward tracing is the exponential nature of knowledge diffusion (Chen and Hicks, 2004). Even a small number of research projects or articles, traced over a long period of time, can create a large amount of data that must be analyzed to identify significant outcomes. For example, in one study, an initial cohort of only 29 papers was cited by 731 unique second-generation papers (“unique” meaning second-generation papers that were not in the initial cohort), which were cited by 9,376 unique papers in the third generation (Hanney et al., 2005). There are currently no standard procedures or best practices to perform the data reduction and other processing necessary to identify significant outcomes or intermediates of interest that might be found among the large base of knowledge flowing from a particular portfolio of research. In this respect, the current state of the art is analogous to the ever-increasing volume of genomic sequence data, which has driven the need for enhanced bioinformatics tools necessary to analyze it (Batley and Edwards, 2009; Magi et al., 2010).

In this paper, we describe one approach we used to discover whether there are long-term, downstream technological advances to which research supported in the late 1970's and early 1980's by the NIGMS may have contributed. NIGMS administers a large portfolio of grants to support basic research in the biomedical sciences. In the five-year period from 1980 (the first year for which sufficient data are available) through 1984, over 18,000 publications cited support from NIGMS funding. We demonstrate one method by which significant health-related outcomes that are built on this research can be identified. In so doing, we also make some observations on the knowledge diffusion network created. This effort represents an initial attempt to define a replicable workflow that might be applied to other large portfolios of research and used routinely by other agencies and organizations to scan for significant outcomes as they occur.

## Materials and Methods

### Data Sources

**Figure 1:**
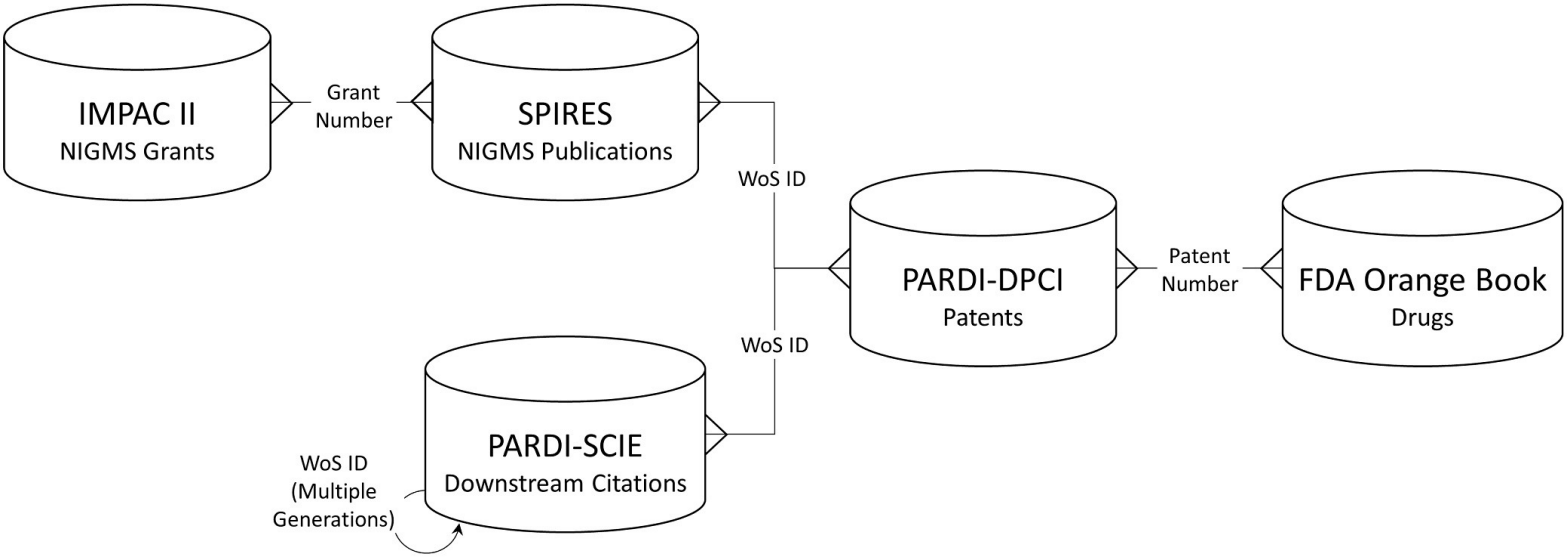
Databases used and linkage keys. IMPAC II, NIH Information for Management Planning Analysis and Coordination; SPIRES, NIH Scientific Publication Information Retrieval and Evaluation System; PARDI-SCIE, Clarivate Science Citation Index Expanded, included in the NIH Portfolio Analysis and Reporting Data Infrastructure; PARDI-DPCI, Clarivate Derwent Patent Citation Index, included in PARDI; WoS, Web of Science.

Long-term outcomes associated with NIGMS-funded research were identified through several types of linked data: NIGMS grants, publications citing NIGMS grant support, “downstream” publications that cited the NIGMS-supported publications, patents whose non-patent literature referenced either an NIGMS-supported or downstream publication, and drug products approved by the U.S. Food and Drug Administration (FDA) that are protected by one or more of the linked patents. Figure 1 shows the data sources used and the structure of the network created among them.

Each of these data sources has certain weaknesses that could prevent a comprehensive and statistically accurate assessment of NIGMS-funded research outcomes. It is known that authors don't always acknowledge their grant support in the papers they publish (in the past, some journals have not permitted such acknowledgments) and the extent of such underreporting is not known. (The ability to link publications to NIH grant support has improved in recent years; in 2008, NIH began requiring reporting of grant-supported publications as a precondition for continued support.) Furthermore, when grant support is cited, it is prone to errors such as typographical mistakes in grant numbers. Similarly, references to non-patent literature in patents is prone to error, as these references are sometimes not detailed enough to uniquely identify the cited paper—for example, only an author and year of publication might be cited. Nor do we have complete information on patents associated with FDA-approved drug products. Of the 6,843 products named in the FDA Orange Book, patent information was available for only 16 percent. However, our primary goal in analyzing these datasets was not to generate a precise and reliable quantitative measurement of research outcomes, but rather to discover long-term outcomes that could be traced back to NIGMS-funded research, as the data allowed, and to enumerate any linkages found.

#### NIH Grants

Information on NIGMS grants was drawn from NIH's Information for Management Planning Analysis and Coordination (IMPAC) II database, an internal NIH database of grant applications and awards maintained by NIH's Office of Electronic Research Administration. While we used an internal database as our source data, a public version of the database is available (National Institutes of Health, 2017a, 2020a).

#### NIGMS Publications

The publications citing NIGMS grant support were retrieved from NIH's Scientific Publication Information Retrieval and Evaluation System (SPIRES). SPIRES is an internal database maintained by NIH's Office of Research Information Systems that relies on the Grant Support tag (GR) in MEDLINE/PubMed publication records (National Library of Medicine, 2019) to link publications to NIH grants. While we used this internal database for this study, a public version is available (National Institutes of Health, 2017b). Publications in SPIRES date from 1980. For this study, all publications from 1980–1984 citing support from NIGMS were selected as the starting point for the analysis.

#### Downstream Publications

Information on “downstream” publications—articles that have cited NIGMS publications—was obtained from NIH's Portfolio Analysis and Reporting Data Infrastructure (PARDI), a non-public NIH database that includes records from the Clarivate Analytics Science Citation Index Expanded® (SCIE). A recursive search of the SCIE can be performed to produce multiple generations of citations. All papers in the SCIE published from 1980 through 2016 were included in the analyses.

#### Patent Awards

Patents that include NIGMS and downstream publications in their non-patent literature references were also obtained from NIH's PARDI, which includes the Clarivate Analytics Derwent World Patents Index®.

#### Drug Products

Patent information on drug products was obtained from FDA's Approved Drug Products with Therapeutic Equivalence Evaluations (Orange Book) Data Files (U.S. Food Drug Administration, 2020). First published in 1980, the “Orange Book” identifies all currently marketed drug products approved on the basis of safety and effectiveness by the FDA. The February 2019 version of the Orange Book was used in the analyses. At that time, there was a total of 6,843 drug products with distinct trade names in the Orange Book. Patent information was available for 1,079 of these products.

### Results

#### The Knowledge Diffusion Network

Previous research has suggested the diffusion of knowledge underlying scientific progress is captured best by multiple generations of citations (Hu et al., 2011). However, we found little in extant literature to guide our choice of how many generations to include in our analysis. The degree and type of impact properly attributable to research when its influence is exerted indirectly through multiple generations of citations is not clear. There are several characteristics that could affect the number of generations that should be included to assess impact and the need for more research on this topic has been noted (Fragkiadaki and Evangelidis, 2016). We found examples of previous research using four to six generations of publications to trace the long-term impact of biomedical research (Grant et al., 2000, 2003; Jones and Hanney, 2016).

In this study we traced the first generation of NIGMS-funded articles forward for two subsequent generations of literature citations to find links to patents. We expected few articles in the first generation of NIGMS-supported publications to be directly cited by patents; the mission of NIGMS is to support research into fundamental biological processes. The Institute does not fund research directly related to a specific disease, life stage, population, or organ system—research which is supported by the other “categorical” NIH institutes and centers (National Institute of General Medical Sciences, 2019b). We considered it more likely that the role of NIGMS research in patented inventions would be found through later generations of research articles that built upon and cited NIGMS-funded research. However, as more generations of publications are added to the network, the relevance of the original NIGMS-funded research to any patent citing that literature may become more tangential. To focus on those patents to which NIGMS research may have contributed most directly, we limited the citation network to only three generations. In previous research, three generations have been considered sufficient to illustrate the usability and feasibility of various measures of impact (Fragkiadaki and Evangelidis, 2016).

**Figure 2:**
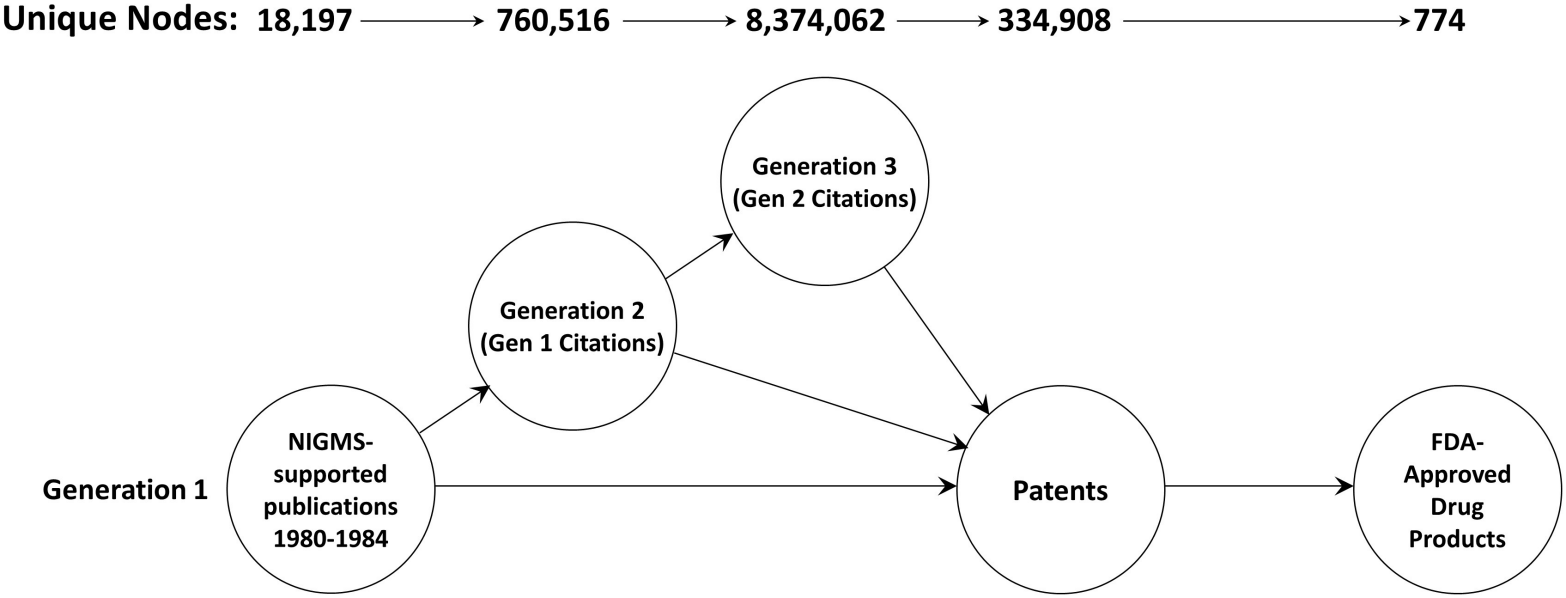
The three-generation publication network. This is a forward trace of 18,197 publications in 1980–1984 citing support from NIGMS.

A total of 18,197 articles published in 1980–1984 cited support from NIGMS (Figure 2). The second generation consisted of 760,516 unique papers and a third generation of 8,374,062 articles cited one or more of the second-generation papers. A total of 334,908 different patents cited at least one article from these three generations of papers. There were 774 different drug products that claimed protection from these patents, representing 11.3 percent of the 6,843 unique trade-named drug products and 71.8 percent of the 1,078 products having patent information in the Orange Book.

#### Citing Publications

**Figure 3:**
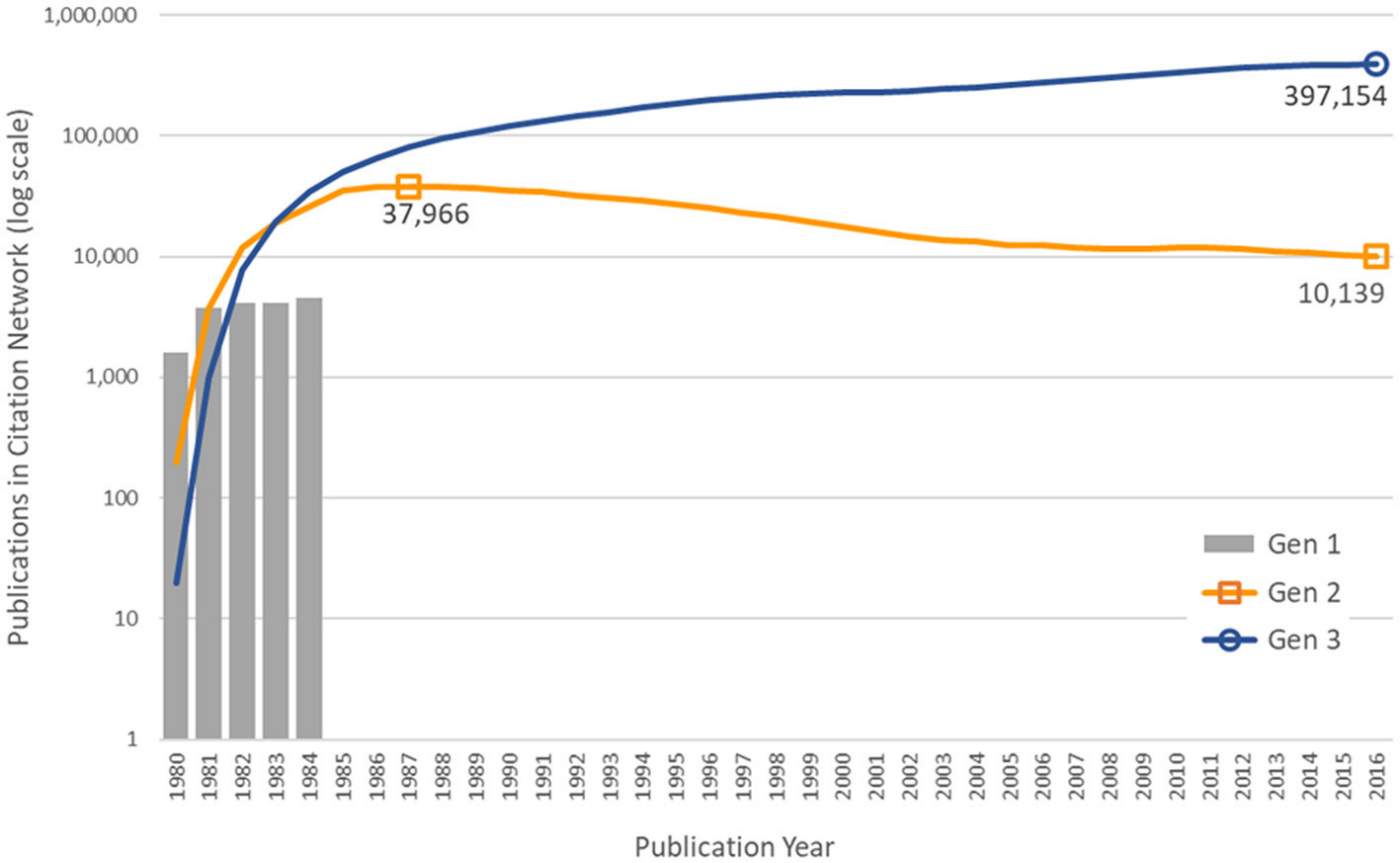
Number of publications in the citation network, by generation and year of publication.

The numbers of publications by year and generation number are shown in Figure 3. As discussed above, the first generation consists of 18,197 articles published in 1980–1984 citing NIGMS support. The number of articles citing the first generation each year reached a peak of 37,966 in 1987, an average of 4.65 years after the NIGMS papers were published. In 2016, the first-generation NIGMS papers were still being cited over 10,000 times. The third-generation papers had not peaked by 2016, when there were 397,154 articles citing one or more the 760,516 papers in the second generation.

#### Citing Patents

Table 1 shows the number of publications in each generation that were cited in patents' non-patent literature. Of the 1980–1984 publications citing NIGMS support, 17.58 percent were cited by at least one of 16,452 patents. The cited publications were referenced in an average of about eight patents.

**Table 1:** Number of publications in each generation cited by patents.

**Generation**	**Publications**	**Cited by patents**	**% Cited**	**Citing patents**	**Patent-pub pairs**	**Avg citations**
1	18,197	3,199	17.58	16,452	26,030	8.14
2	760,516	127,132	16.72	159,378	869,666	6.84
3	8,374,062	673,977	8.05	319,481	3,473,972	5.15
Total	9,152,775	804,308	8.79	334,908	4,369,668	5.43

*Average citations calculated using only publications cited by at least one patent*.

**Figure 4:**
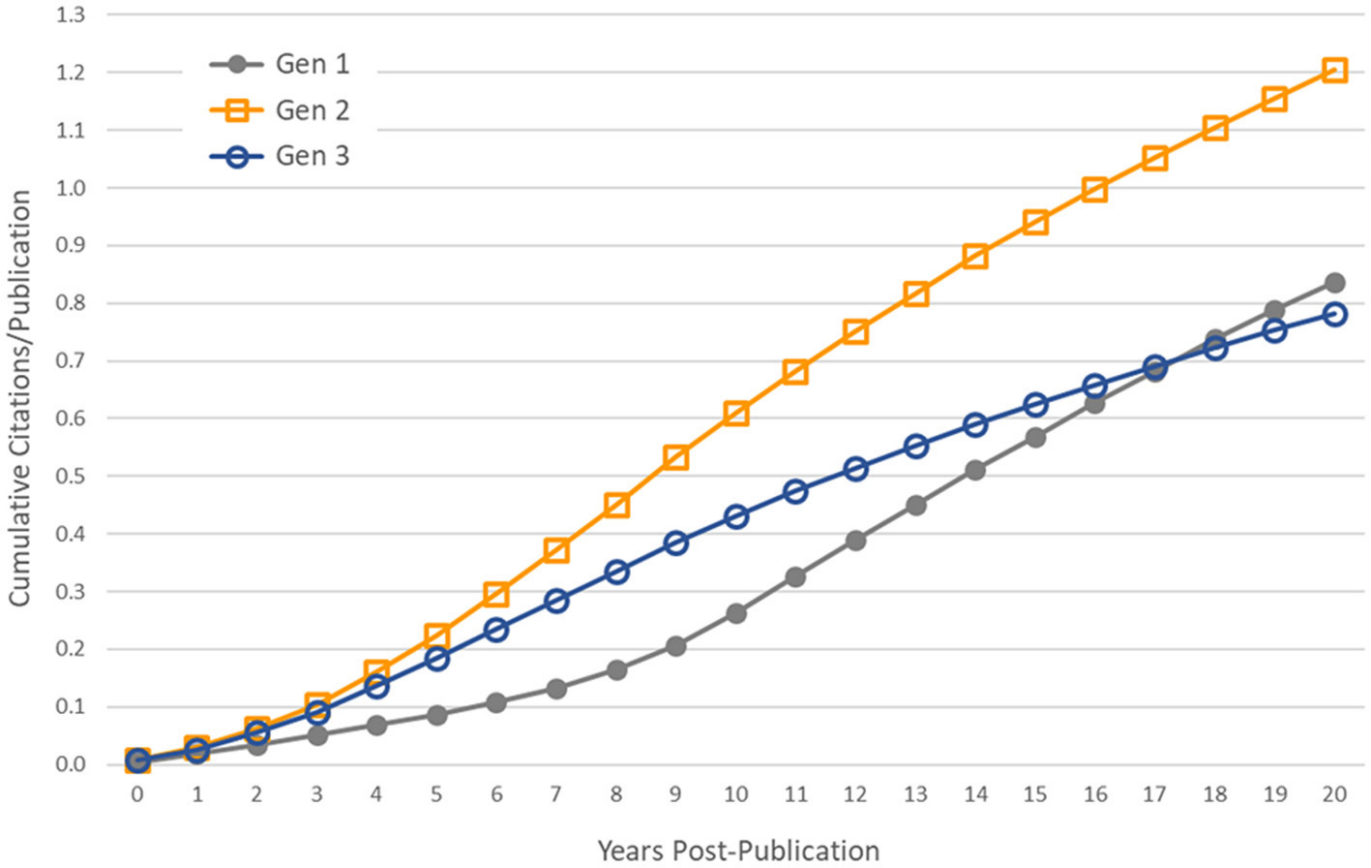
Controlling for censored time series data. Average cumulative number of patent citations to papers published in the year 1993 and earlier, by generation.

Subsequent generations of publications were less likely to be cited by a patent, and those papers that were cited were referenced on fewer patents. However, these statistics are influenced by the censored distributions of the second and third generations of articles. Many of these articles have been published in recent years and some will be cited by patents in the future. To control for this effect, we used only papers published in the year 1993 and earlier—providing a citation follow-up time of at least 20 years for all papers—and calculated the average cumulative number of patent citations that papers received in the first 20 years post-publication. These cumulative distribution functions are shown in Figure 4. In general, the first generation of papers, which we expect to be more heavily weighted toward basic research, have fewer patent citations in the years immediately following publication, but they are cited at a higher rate over longer periods of time than second- and third-generation papers, eventually surpassing generation 3.

#### Time to First Patent Citation

**Figure 5:**
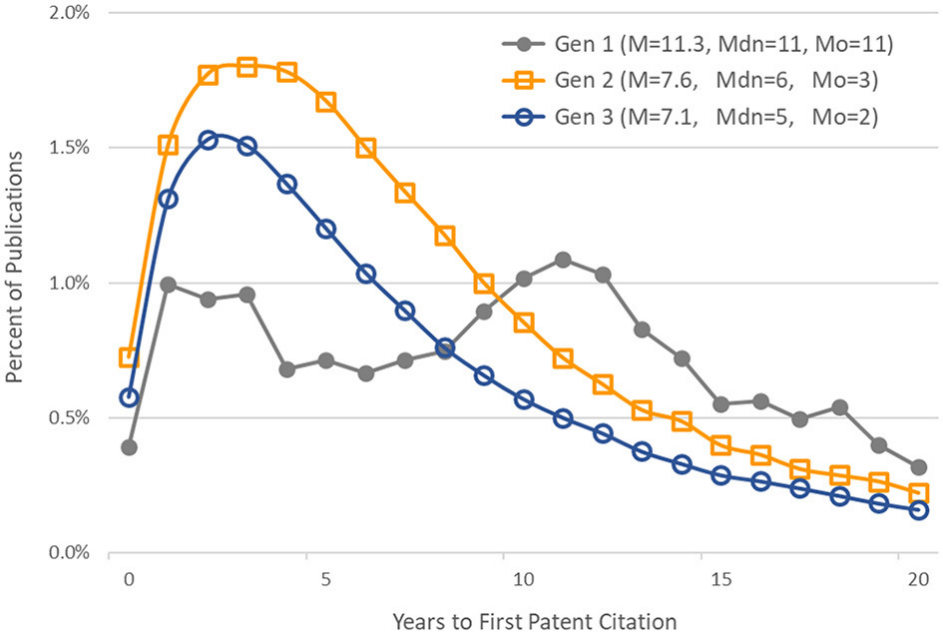
Distribution of publications by time to first patent citation. Generation 1 articles published in 1980–1984. Generations 2 and 3 articles published in 1980–1993. Also noted are the mean (M), median (Mdn), and mode (Mo) of each generation's distribution.

Figure 5 shows, for each generation, the distribution of publications by number of years to first patent citation. The mean (M), median (Mdn), and mode (Mo) are also given for each generation's distribution.

#### Linked Products

Of the 774 products linked to patents in the NIGMS citation network, six (shown in Table 2), were among the top 20 best-selling drugs in the U.S. for 2018 (Questex, 2020). The total sales of these six products was $19.52 billion. “Publication Generation” indicates the generation in which these products' patents entered the citation network.

**Table 2:** U.S. sales in 2018 for top-selling drugs that were linked to NIGMS-supported research.

**Rank**	**Trade_name**	**Publication generation**	**Sales ($B)**
8	Imbruvica	2	4.10
12	Genvoya	3	3.63
13	Lyrica	3	3.59
16	Ibrance	3	2.90
19	Victoza	2	2.70
20	Truvada	2	2.60

#### Data Reduction

This high-level summary of the citation network provided us with descriptive information on the broad diffusion of knowledge developed through NIGMS-funded research. However, our primary interest is in how to distill this large amount of information to identify *specific* outcomes of interest and significant events in the research and development process. We turned to the network analysis and visualization platform Gephi (Bastian et al., 2009) to analyze the network and locate nodes of significance.

**Figure 6:**
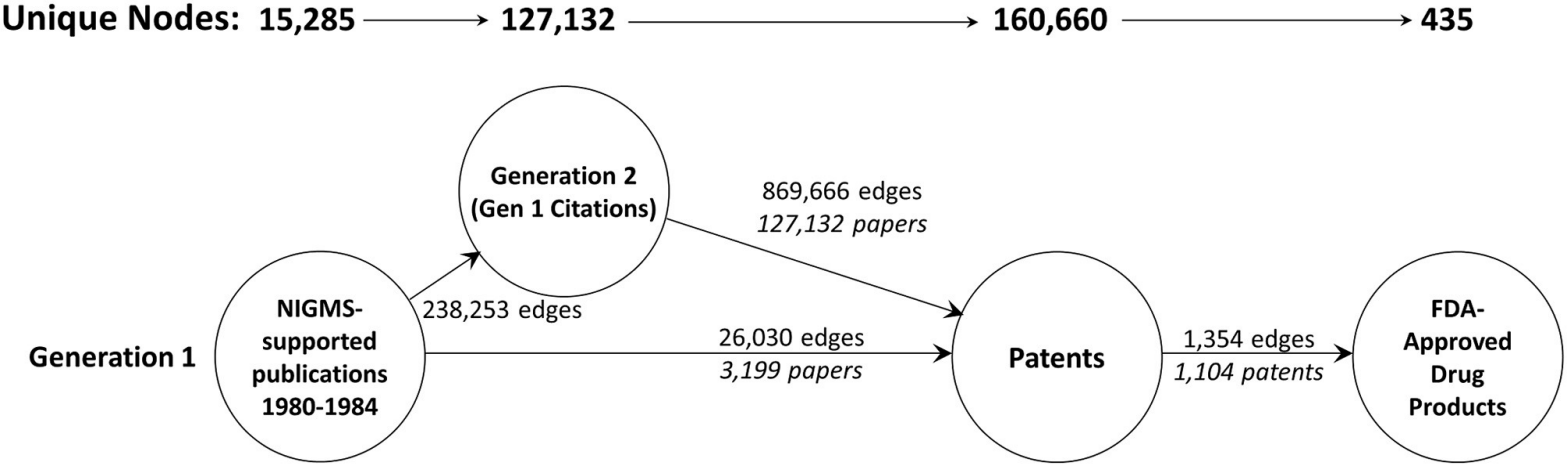
The two-generation publication network. A forward trace of 15,285 publications in 1980–1984 citing support from NIGMS that eventually led to one or more patents.

Unfortunately, a network of the size we originally created, with 9.5 million nodes, exceeds the capacity of Gephi (as well as some other popular graph visualization and analysis tools; Pavlopoulos et al., 2017). As a result, we included only two generations of publications—the original set of 18,197 articles supported by NIGMS and all the papers that cited one or more of these NIGMS publications—and, of these, only publications eventually led to a patent. The numbers of nodes in this reduced network and edges are shown in Figure 6. The NIGMS publications were ultimately linked to 435 drug products, representing 6.4 percent of the 6,843 unique trade-named drug products and 40.4 percent of the 1,078 products having patent information in the Orange Book.

**Figure 7:**
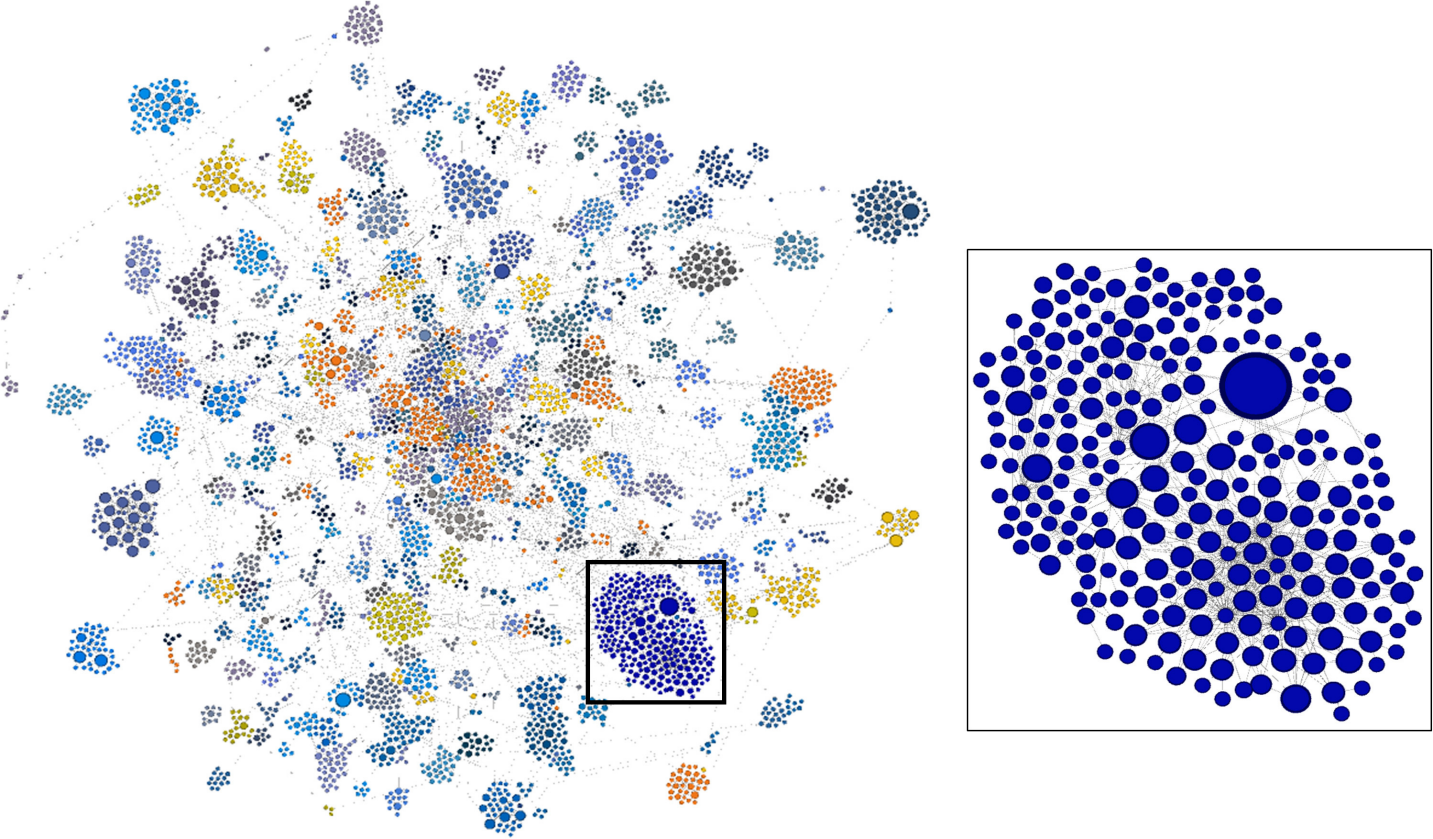
Complete two-generation network as a directed graph. Highly linked clusters are identified by color. Blue cluster in box is enlarged at right. Visualization by Gephi.

A visualization of this network as a directed graph is shown in Figure 7. To discern meaningful relationships or patterns in the network, we identified clusters of related research, patents, and products using the Louvain Method of community detection (Blondel et al., 2008), implemented in Gephi using a randomized parameter, no weights, and 1.0 resolution. Network visualization was performed with Gephi 0.9.1 using the ForceAtlas2 layout algorithm and default parameters.

**Figure 8:**
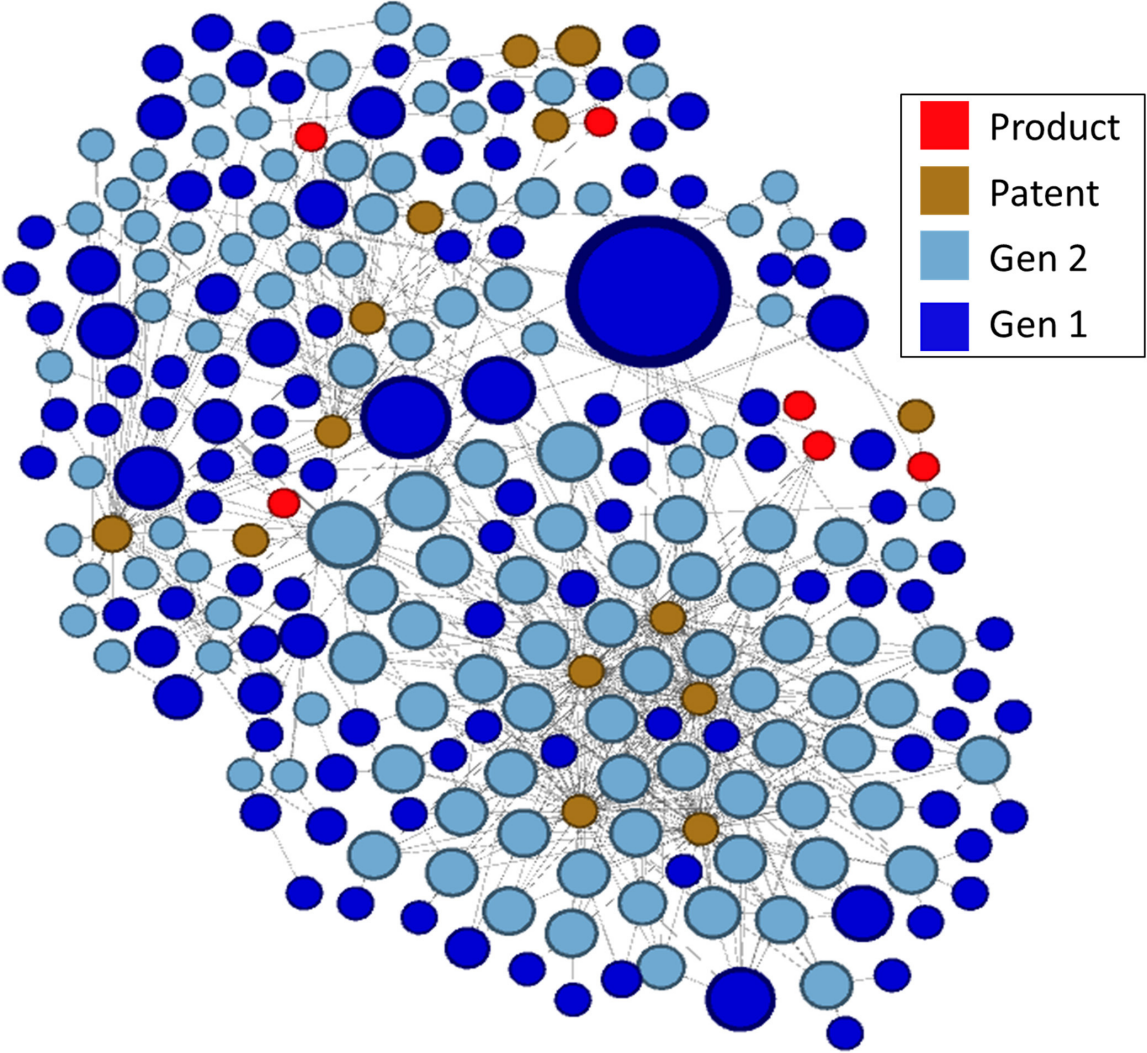
Highly linked cluster shown in Figure 7, by type of node.

In the lower right portion of the network in Figure 7 is a cluster that appears to be particularly large and whose nodes are highly linked. We arbitrarily selected this cluster for further analysis. In Figure 8, this cluster is isolated and color-coded by node type, making it easier to identify patents and drug products that are linked to the outputs of research supported by NIGMS. The size of the blue publication nodes is proportional to the number of times each has been cited by other publications or patents (indegree). The size of the brown patent nodes is also proportional to their indegree, the number of drug products for which they provide intellectual property protection.

#### Developing a Product Trace

There were 14 patents in the cluster, related to six drug products, all of which affect gene expression, including several oligonucleotide therapeutics, a relatively new class of drugs made of chemically synthesized nucleic acids (Smith and Zain, 2019):

Cubicin® RF (daptomycin) is a last-resort antibiotic with excellent activity against Gram-positive pathogens. It was first approved by the FDA for use in the treatment of skin infections. It has a distinct mechanism of action causing rapid depolarization of membrane potential, disrupting cell membrane function to inhibit protein, DNA, and RNA synthesis;

Epiduo® (adapalene and benzoyl peroxide) is a treatment for severe acne. Adapalene binds to retinoic acid nuclear receptors, which act as transcription factors to regulate the expression of mRNA for proteins modulating cell differentiation and keratinization;

Kynamro® (mipomersen) is an adjunct to lipid-lowering medications to reduce LDL in patients with homozygous familial hypercholesterolemia. It is an antisense oligonucleotide targeted to human messenger ribonucleic acid (mRNA) for apo B-100, the principal apolipoprotein of LDL;

Onpattro® (patisiran) is a small interfering RNA (siRNA) oligonucleotide for the treatment of polyneuropathy in people with hereditary transthyretin-mediated amyloidosis. It is the first siRNA-based drug approved by the FDA;

Tegsedi™ (inotersen) is for the treatment of polyneuropathy in people with hereditary transthyretin-mediated amyloidosis. It also is an antisense oligonucleotide that inhibits hepatic production of transthyretin by binding to mRNA;

Zemdri™ (plazomicin) is an aminoglycoside antibacterial for the treatment of complicated urinary tract infections. It acts by binding to bacterial 30S ribosomal subunits, interfering with mRNA and protein synthesis.

**Figure 9:**
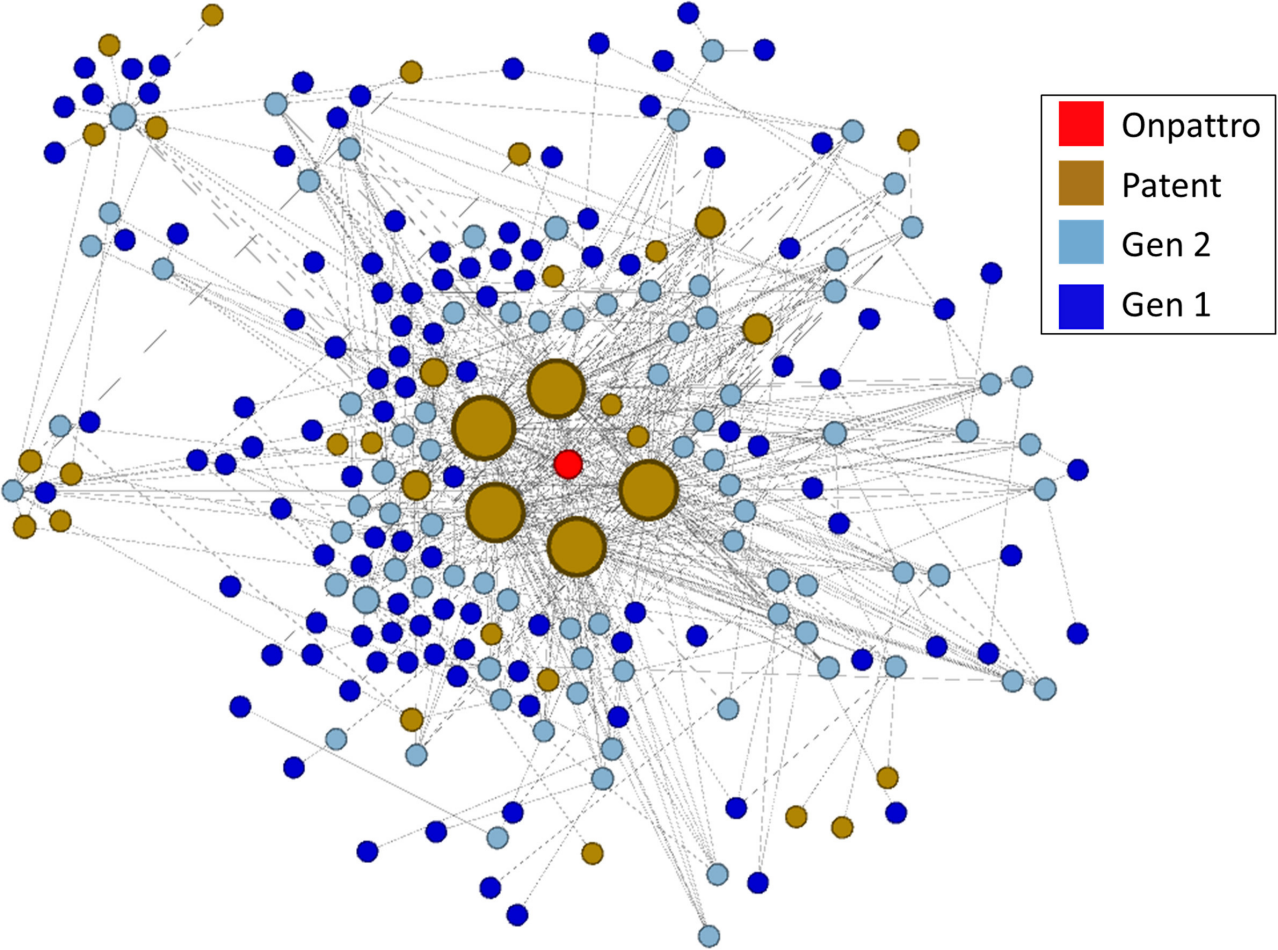
Patents and publications in the Onpattro cluster. Seven patents are associated with Onpattro in the FDA Orange book. The cluster also contains seven additional patents not related to Onpattro.

The linkage of NIGMS-funded research to Onpattro, being the first siRNA-based drug and only recently approved for use, was a particularly interesting discovery. We reduced the data further by examining the nodes in the immediate neighborhood of Onpattro. This smaller network is shown in Figure 9, where the nodes have been resized according to their indegree within the Onpattro network. The single product node, Onpattro, is colored red and located in the center. It is surrounded by seven patents, in brown, linked to NIGMS-funded research that are associated with Onpattro in the FDA Orange Book (out of a total of 21 patents for Onpattro). These seven patents fall into three families (Table 3). In the Government Interest section of one of these patent families, *RNA sequence-specific mediators of RNA interference*, support is acknowledged from NIGMS grant number GM034277, a grant on the regulation of mRNA processing awarded to Philip Sharp, a Nobel Laureate who co-discovered RNA splicing.

**Table 3:** Onpattro patent families and number found in the NIGMS network.

**Patent family**	**Patent year(s)**	**Patents in family**	**Patents in NIGMS network**	**Government interest**
Lipid formulations for nucleic acid delivery	2011–2016	4	0	
Lipid formulation	2012, 2014	2	0	
Nuclease resistant double-stranded ribonucleic acid	2012	1	0	
Compositions and methods for inhibiting expression of transthyretin	2012–2016	3	2	
RNA sequence-specific mediators of RNA interference	2013, 2015	2	2	GM034277
RNA interference mediating small RNA molecules	2013–2017	6	3	
Lipid containing formulations	2014	1	0	
2′-methoxy substituted oligomeric compounds and compositions for use in gene modulations	2018	2	0	

*Onpattro patents from U.S. Food Drug Administration (2020)*.

The five patents with the largest indegree (i.e., largest number of connections to the NIGMS publication network), evident in Figure 9, are from two families: *RNA sequence-specific mediators of RNA interference* and *RNA interference mediating small RNA molecules*. The periphery of Figure 9 also shows seven patents citing literature in the Onpattro network but which are not themselves linked to Onpattro in the Orange Book.

**Figure 10:**
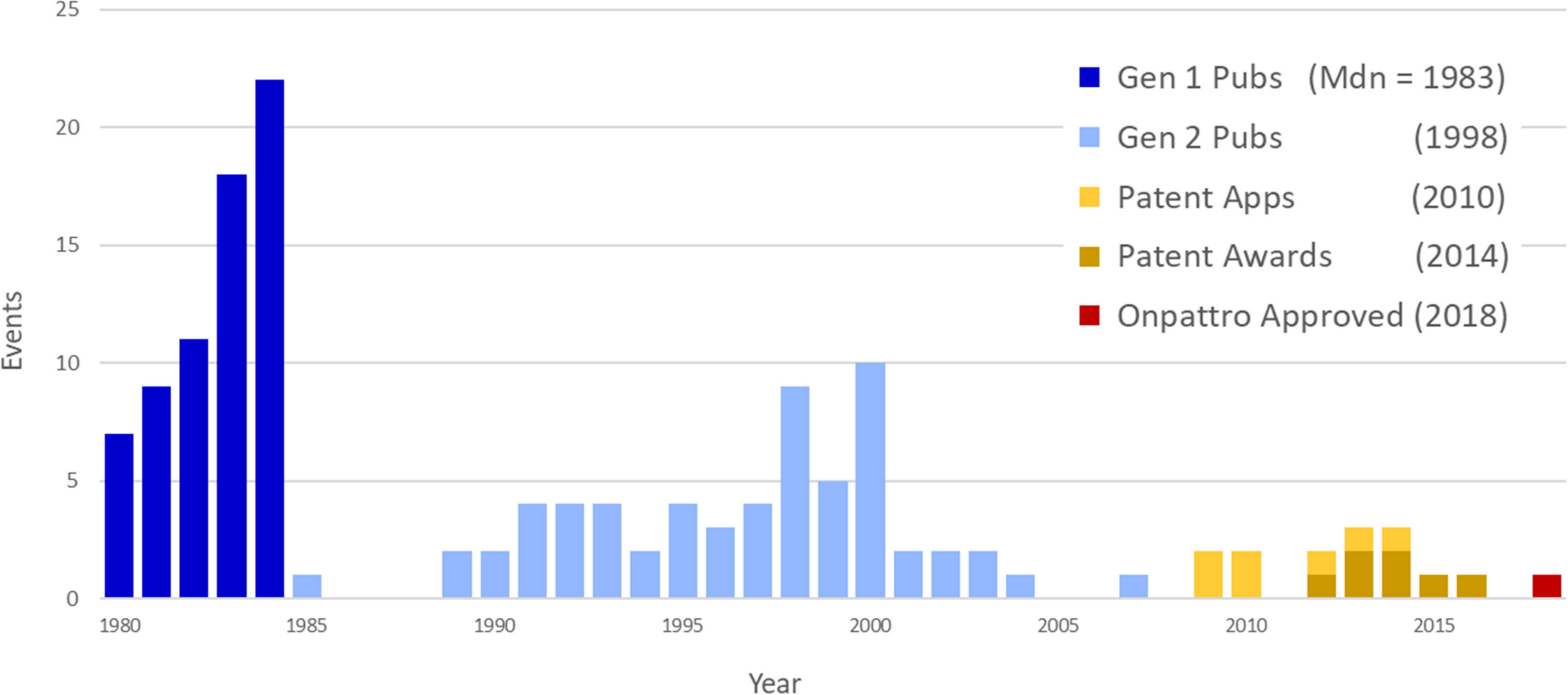
Timeline of events in the Onpattro development network.

To establish a chronology of events involved in the development of Onpattro, the network nodes were placed on a timeline using publication years, patent application and award dates, and the approval of Onpattro in 2018 (Figure 10). The timeline includes the network's 67 first-generation publications in 1980–1984 (median publication year = 1983), 62 second-generation publications (median publication year = 1998), and the seven patent applications (median date = 2010) and awards (median date = 2014). Of the 62 second-generation publications, 19 resulted from NIGMS-funded research. Across both generations, NIGMS supported 86 (66.7 percent) of the 129 publications in the Onpattro network.

#### NIGMS Grant Support Linked to Onpattro Development

Finally, we identified the specific NIGMS-funded basic research that produced many of the articles in the Onpattro network. A total of 80 NIGMS grants were cited by publications in the Onpattro network. Ten of these were responsible for generating 32 first- and second-generation articles (Table 4) representing 37 percent of all NIGMS-supported publications and almost one-quarter of all articles in the network. Eight of these grants generated 34.3 percent of the first-generation publications from which the knowledge dissemination network was developed. Two of the ten grants were awarded after 1984 and supported only publications in the second generation. All of these grants involve well-known investigators, including three Nobel Laureates, working in areas of basic research critical to the development of oligonucleotide therapeutics and other drugs affecting gene expression. Also shown in Table 4 is the total amount of NIGMS funding for these grants through 1984, the final year of the first-generation publications used to generate the Onpattro network. Total NIGMS support for the first-generation publications was $206 million.

**Table 4:** NIGMS research grants generating 37 percent of all NIGMS-supported publications associated with Onpattro development.

**Investigator**	**Gen1 pubs**	**Gen2 pubs**	**Grant**	**Grant title**	**First funded**	**Funding through 1984**
Fire, Andrew	0	7	GM037706	Gene regulation during early development of C elegans	1987	N/A
Horvitz, H Robert	5	1	GM024663	Genetic analysis of nematode egg-laying	1978	$28,497,885
			GM024943	control of cell division in the nematode C elegans	1978	$29,721,139
Apirion, David	5	0	GM019821	Genetics and biochemistry of RNA processing in *E. coli*	1974	$27,066,386
			GM025890	The molecular biology of RNA turnover in *E. coli*	1979	$10,185,015
Hershey, John	4	0	GM022135	Mechanism of initiation of protein biosynthesis	1975	$30,604,835
Levin, Daniel	3	0	GM024825	Control of protein synthesis by double-stranded RNA	1978	$29,321,169
T'so, Paul	3	0	GM016066	Nucleic acid chemistry and its biomedical application	1970	$19,557,228
Turner, Douglas	3	0	GM022939	Kinetic and spectroscopic studies of nucleic acids	1976	$30,598,690
Sharp, Philip	0	1	GM034277	Regulation of MRNA processing	1985	N/A
Total	23	9				$205,552,347

### Discussion

This initial attempt to interrogate a large network of documentary evidence, beginning with the results of basic research funded by NIGMS in 1980–1984, provided us with several interesting findings to be explored in more depth. The knowledge flowing from this body of research was traced to the development of 774 drug products, including some of the most popular drugs in use today. Six of these drugs were among the best-selling in 2018, with sales of $19.5 billion (Questex, 2020). In comparison, the total funding for the 48 research grants producing this research, through 1984, was $82.9 million, ~311.7 million in 2018 dollars (National Institutes of Health, 2020c). Limiting grant funding to the five years preceding publication—research in the year of publication and the four prior years—reduces the total NIGMS investment that gave rise to this knowledge diffusion network to $44 million, ~$165 million in 2018 dollars.

We lack sufficient data to calculate a return on investment (ROI) from these figures. A proper calculation of ROI would require more completely identifying all non-NIGMS inputs contributing to long-term outcomes, including negative outcomes (i.e., revenue losses), applying an appropriate economic valuation to the outcomes, and weighing the attribution of outcomes to each input (Buxton et al., 2004). Previous studies have more rigorously produced estimates of the economic returns of funding for health-related research (Buxton et al., 2004; RAND Europe., 2008; Grant and Buxton, 2018). We will simply note that, while NIGMS funding for basic research is only one portion of the total required to bring these products to market, these fundamental discoveries were critical to drug development and the amount of NIGMS funding required was small relative to the value of the outcomes to which they contributed.

We were also able to gain a better sense of the amount of time required for the diffusion of the basic research findings generated by NIGMS funding. Second-generation citations of research published in 1980–1984 peaked in 1987, an average of 4.65 years after publication [consistent with previous findings, see Fukuzawa and Ida (2016)], but there were still many citations of this work in 2016, more than thirty years later. It was also interesting to see that third-generation citations of this research had not peaked by 2016 and continue to grow in number.

Carpenter et al. (1980) found that patents cite relatively recent literature, but these citations varied by technology area; the median “age” of articles cited (the time elapsed from the paper's publication to its patent citation) by gas laser patents was only three years. It was slightly longer in prostaglandin patents. Chen and Hicks (2004), studying tissue engineering research articles, found the time elapsed between papers' publication year and their first front-page citation in a patent had a mean of 9.6 years, and a mode of 2 years. While patents may more frequently cite recent research, it is not necessarily the case that most of a research article's patent citations will occur shortly after publication.

In our study, the time elapsed from an article's publication and its first citation in a patent application varied as a function of a publication's generation in the citation network. We provided a minimum of 20 years follow-up for articles in all generations. As expected, the time elapsed from the original basic research funded by NIGMS to its first citation in a patent was longer than for later generations of publications that built on this research. The median time to first patent citation in the first generation of 1980–1984 articles was 11 years. The lags for the second- and third-generation papers were shorter, at 6 and 5 years, respectively. The lag distributions for generations 2 and 3 were similar to those found by Chen and Hicks (2004).

Using clustering techniques (which required that we use a smaller dataset excluding third-generation citations), we were able to discover meaningful clusters of papers, patents, and products. One cluster, selected arbitrarily, was a network of documents related to a class of drugs affecting gene expression, including several recently-developed oligonucleotide therapeutics. We were able to identify the specific NIGMS research grants and investigators whose research contributed to the development of one of these drugs, Onpattro. From the network of linked documents, we were able to chart key events occurring over a 38-year period from the first NIGMS-supported publications in 1980-1984 to approval of Onpattro in 2018. About one-half of the NIGMS-supported articles were produced by grants totaling about $206 million through 1984. It is not clear how much of this funding would have directly contributed to these key publications; for example, some of these grants began in the early 1970s. By contrast, net product revenues for Onpattro through 2019 were $179 million and sales of $285 to $315 million are forecast for 2020 (Alnylam, 2020).

The Onpattro example demonstrates the ability to easily discover useful new knowledge from large linked datasets of information. The automated procedures to do so may provide a useful alternative to the labor-intensive approaches that have been used in the past, such as that described in Comroe and Dripps (1976). While the validity of their findings has been challenged (Smith, 1987; Grant et al., 2003), we cite Comroe and Dripps only to exemplify the effort that can be required by this type of study. The Comroe and Dripps study employed about 200 consultants to identify the most significant advances in cardiovascular and pulmonary medicine, the essential bodies of knowledge required for these advances, and key articles in these knowledge flows. They used the expert opinion of many consultants to avoid bias in the selection of key prior research and articles. More recent tracing studies have continued to rely on labor-intensive methods (Smith, 1987; Contopoulos-Ioannidis et al., 2003; Mayernik et al., 2016). We employ a different approach to avoiding bias by using objective linkages among documentary evidence, and our study also employed a single primary researcher with access to the necessary databases and analytic tools.

Just as Chen and Hicks' work was the start of a long-term program to develop new analytic methods capturing knowledge diffusion (Chen and Hicks, 2004), we view our study as an exploratory effort to assess the utility of linked databases in tracing the long-term influence of a program of basic research in the biomedical sciences. Our ability to link new technologies to NIGMS-funded research was facilitated by an existing data infrastructure (PubMed) that links publications to NIH grants in the biomedical sciences (National Library of Medicine, 2019). Such resources are beginning to be made available in more areas of science. Other agencies, funding research in diverse fields, have started to make publications and other research products associated with their grants available through public data sources such as Federal RePORTER, an online searchable database developed by STAR METRICS, a consortium of US science agencies (Onken, 2016). Beginning in 2019, the EU has made project-linked publications available through its Open Data Portal (European Commission, 2020) and the UK provides data on publications linked to projects funding by nine agencies funding research in a range of fields (UK Research Innovation, 2020). Commercial bibliographic data services such as Web of Science, SCOPUS, and Dimensions have been capturing such information from funding acknowledgments in papers for some time (Rigby, 2011; Hook et al., 2018). Even in the absence of project identifiers linked to publications, our procedures allow the search for long-term outcomes to begin with any set of publications produced by a portfolio of research.

Procedures like those we describe here offer objective, reliable, and less time-consuming ways to discover knowledge flows contributing to new technologies and the research playing a critical role (van Raan, 2017). Our approach is, however, only one of many possible approaches. More research is needed to find other, more optimal approaches for linking databases, identifying critical nodes in knowledge flows, and exploring the meaningfulness of the networks discovered. Greater understanding is needed of the degree and type of impact properly attributable to research when its influence is exerted indirectly through multiple generations of citations. This will require more in-depth study that builds on the initial effort presented in this paper. For example, previous research has demonstrated a shift from basic to clinical science across forward generations of citations (Grant et al., 2000, 2003). If corroborated using the citation network developed in our study, we might be able to describe with greater specificity the contributions made by basic research. By investing in such research, automated procedures thus developed can be quickly and easily applied to other research programs, significantly reducing the time and effort required to demonstrate, in an objective way, long-term contributions of the results flowing from basic research programs.

### Data Availability Statement

Publicly available datasets were analyzed in this study. The first generation of NIGMS-supported publications and grant numbers, which formed the foundation of the citation network created in this paper, is publicly available at https://doi.org/10.6084/m9.figshare.12671045. Subsequent generations of publications in the citation network were obtained from the proprietary Clarivate Web of Science database and our license restricts distribution. The FDA Orange Book data used in this study can be found here: https://www.fda.gov/drugs/drug-approvals-and-databases/orange-book-data-files.

## Ethics Statement

Written informed consent was obtained from the individual(s) for the publication of any potentially identifiable images or data included in this article.

## Author Contributions

JO designed the study, performed data analysis, and wrote the initial draft manuscript. AM assisted in conceptualizing the study and revised the manuscript critically. RA secured funding, oversaw data interpretation, and revised the manuscript critically. All authors read and approved the final manuscript.

## Conflict of Interest

The authors are employed by, or receive contract support from, the National Institute of General Medical Sciences, which funded all aspects of this study.

## References

[B1] Alnylam (2020). Alnylam Pharmaceuticals Reports Fourth Quarter and Full Year 2019 Financial Results and Highlights Recent Period Activity. Available online at: https://investors.alnylam.com/press-release?id=24491 (accessed February 19, 2020).

[B2] Bastian M. Heymann S. Jacomy M. (2009). Gephi: an open source software for exploring and manipulating networks, in Third International AAAI Conference on Weblogs and Social Media (San Jose, CA), 361–362.

[B3] Batley J. Edwards D. (2009). Genome sequence data: management, storage, and visualization. Biotechniques 46, 333–336. 10.2144/000113134 19480628

[B4] Blondel V. D. Guillaume J-L, Lambiotte, R. Lefebvre E. (2008). Fast unfolding of communities in large networks. *J. Stat. Mech*. Theory Exp. 10, 10008–10019. 10.1088/1742-5468/2008/10/P10008

[B5] Buxton M. Hanney S. (1996). How can payback from health services research be assessed? J. Health Serv. Res. Policy 1, 35–43. 10.1177/135581969600100107 10180843

[B6] Buxton M. Hanney S. Jones T. (2004). Estimating the economic value to societies of the impact of health research: a critical review. Bull. World Health Organ 82, 733–739. 15643793 PMC2623029

[B7] Carpenter M. P. Cooper M. Narin F. (1980). Linkage between basic research literature and patents. Res. Manage. 23, 30–35. 10.1080/00345334.1980.11756595

[B8] Centers for Disease Control Prevention (2017). About the Science Impact Project. Available online at: https://www.cdc.gov/od/science/impact/about.html (accessed February 3, 2020).

[B9] Chen C. Hicks D. (2004). Tracing knowledge diffusion. Scientometrics 59, 199–211. 10.1023/B:SCIE.0000018528.59913.48

[B10] Comroe J. H. Dripps R. D. (1976). Scientific basis for the support of biomedical science. Science 192, 105–111. 10.1126/science.769161 769161

[B11] Contopoulos-Ioannidis D. G. Ntzani E. E. Ioannidis J. P. A. (2003). Translation of highly promising basic science research into clinical applications. Am. J. Med. 114, 477–484. 10.1016/S0002-9343(03)00013-5 12731504

[B12] Donovan C. (2007). The qualitative future of research evaluation. Sci. Public Policy 34, 585–597. 10.3152/030234207X256538

[B13] Donovan C. (2011). State of the art in assessing research impact: introduction to a special issue. Res. Eval. 20, 175–179. 10.3152/095820211X13118583635918

[B14] Donovan C. Hanney S. (2011). The “payback framework” explained. Res. Eval. 20, 181–183. 10.3152/095820211X13118583635756

[B15] European Commission (2020). EU Open Data Portal: Access to European Union Open Data. Available online at: https://data.europa.eu/euodp/en/home (accessed July 8, 2020).

[B16] Fortunato S. Bergstrom C. T. Borner K. Evans J. A. Helbing D. Milojevic S.. (2018). Science of science. Science 359, 1007–1014. 10.1126/science.aao0185 29496846 PMC5949209

[B17] Fragkiadaki E. Evangelidis G. (2016). Three novel indirect indicators for the assessment of papers and authors based on generations of citations. Scientometrics 106, 657–694. 10.1007/s11192-015-1802-4

[B18] Fukuzawa N. Ida T. (2016). Science linkages between scientific articles and patents for leading scientists in the life and medical sciences field: the case of Japan. Scientometrics 106, 629–644. 10.1007/s11192-015-1795-z

[B19] Grant J. Buxton M. J. (2018). Economic returns to medical research funding. BMJ Open 8:e022131. 10.1136/bmjopen-2018-022131 PMC6144334 30201795

[B20] Grant J. Cottrell R. Cluzeau F. Fawcett G. (2000). Evaluating “payback” on biomedical research from papers cited in clinical guidelines: applied bibliometric study. BMJ 320, 1107–1111. 10.1136/bmj.320.7242.1107 10775218 PMC27352

[B21] Grant J. Green L. Mason B. (2003). Basic research and health: a reassessment of the scientific basis for the support of biomedical science. Res. Eval. 12, 217–224. 10.3152/147154403781776618

[B22] Hanney S. R. Home P. D. Frame I. Grant J. Green P. Buxton M. J. (2005). Identifying the impact of diabetes research. Diabetic Med. 23, 176–184. 10.1111/j.1464-5491.2005.01753.x 16433716

[B23] Hicks D. Wouters P. Waltman L. de Rijcke S. Rafols I. (2015). Bibliometrics: the leiden manifesto for research metrics. Nature 520, 429–431. 10.1038/520429a 25903611

[B24] Hook D. W. Porter S. J Herzog C. (2018) Dimensions: building context for search evaluation. Front. Res. Metr. Anal. 3:23. 10.3389/frma.2018.00023

[B25] Hu X. Rousseau R. Chen J. (2011). On the definition of forward and backward citation generations. J. Infometr. 5, 27–36. 10.1016/j.joi.2010.07.004

[B26] Husbands Fealing K. Lane J. I. Marburger J. H. Shipp S. S. (2011). Editor's introduction, in The Science of Science Policy: A Handbook, eds Husbands Fealing K. Lane J. Marburger J. III Shipp S. (Palo Alto, CA: Stanford University Press), 1–8.

[B27] Hutchins B. I. Baker K. L. Davis M. T. Diwersy M. A. Haque E. Harriman R. M.. (2019). The NIH open citation collection: a public access, broad coverage resource. PLoS Biol. 17:e3000385. 10.1371/journal.pbio.3000385 31600197 PMC6786512

[B28] Jones T. H. Hanney S. (2016). Tracing the indirect societal impacts of biomedical research: development and piloting of a technique based on citations. Scientometrics 107, 975–1003. 10.1007/s11192-016-1895-4 27340306 PMC4869749

[B29] Kamenetzky A. Hinrichs-Krapels S. (2020). How do organisations implement research impact assessment (RIA) principles and good practice? A narrative review and exploratory study of four international research funding and administrative organisations. Health Res. Policy Syst. 18:6. 10.1186/s12961-019-0515-1 31959198 PMC6971910

[B30] Kearnes M. Wienroth M. (2011). Tools of the trade: UK research intermediaries and the politics of impacts. Minerva 49, 153–174. 10.1007/s11024-011-9172-4

[B31] Keserci S. Livingston E. Wan L. Pico A. R. Chacko G. (2017). Research synergy and drug development: bright stars in neighboring constellations. Heliyon 3:e00442. 10.1016/j.heliyon.2017.e00442 29264408 PMC5727381

[B32] King's College London and Digital Science (2015). The Nature, Scale and Beneficiaries of Research Impact: An Initial Analysis of Research Excellence Framework (REF) 2014 Impact Case Studies. Bristol: HEFCE.

[B33] Magi A. Benelli M. Gozzini A. Girolami F. Torricelli F. Brandi M. L. (2010). Bioinformatics for next generation sequencing data. Genes 1, 294–307. 10.3390/genes1020294 24710047 PMC3954090

[B34] Mayernik M. S. Hart D. L. Maull K. E. Weber N. M. (2016). Assessing and tracing the outcomes and impact of research infrastructures. J. Assoc. Inf. Sci. Tech. 68, 1341–1359. 10.1002/asi.23721

[B35] Narin F. (2013). Tracing the paths from basic research to economic impact. F&M Sci. 2013, 67–88.

[B36] National Academies of Sciences Engineering, and Medicine. (2017). Using Narrative and Data to Communicate the Value of Science: Proceedings of a Workshop—in Brief . Washington, DC: The National Academies Press.

[B37] National Institute of General Medical Sciences (2019a). NIGMS Feedback Loop: Apply to SCISIPBIO: A Joint Initiative Between NIGMS and NSF to Support Research on the Science of Science and Innovation Policy. Available online at: https://loop.nigms.nih.gov/2019/02/apply-to-scisipbio-a-joint-initiative-between-nigms-and-nsf-to-support-research-on-the-science-of-science-and-innovation-policy (accessed April 9, 2020).

[B38] National Institute of General Medical Sciences (2019b). About NIGMS: Overview. Available online at: https://www.nigms.nih.gov/about/overview (accessed July 3, 2020).

[B39] National Institutes of Health (2014). Scientific Management Review Board Report on Approaches to Assess the Value of Biomedical Research Supported by NIH. Available online at: https://smrb.od.nih.gov/documents/reports/VOBR%20SMRB__Report_2014.pdf (accessed January 22, 2019).

[B40] National Institutes of Health (2017a). Computer Retrieval of Information on Scientific Projects (CRISP) Legacy Data. Available online at: https://exporter.nih.gov/crisp_catalog.aspx (accessed February 6, 2020).

[B41] National Institutes of Health (2017b). ExPORTER Data Catalog: Publications. Available online at: https://exporter.nih.gov/ExPORTER_Catalog.aspx?sid=0&index=2 (accessed February 6, 2020).

[B42] National Institutes of Health (2018a). Impact of NIH Research: Our Stories. Available online at: https://www.nih.gov/about-nih/what-we-do/impact-nih-research/our-stories (accessed January 29, 2020).

[B43] National Institutes of Health (2018b). Childhood Hib Vaccines: Nearly Eliminating the Threat of Bacterial Meningitis. Available online at: https://www.nih.gov/sites/default/files/about-nih/impact/childhood-hib-vaccines-case-study.pdf (accessed February 3, 2020).

[B44] National Institutes of Health (2019). Federal Reporter. Available online at: https://federalreporter.nih.gov (accessed January 29, 2020).

[B45] National Institutes of Health (2020a). NIH Research Portfolio Online Reporting Tools: RePORTER. Available online at: https://projectreporter.nih.gov (accessed January 29, 2020).

[B46] National Institutes of Health (2020c). Biomedical Research and Development Price Index (BRDPI). Available online at: https://officeofbudget.od.nih.gov/gbipriceindexes.html (accessed March 11, 2020).

[B47] National Institutes of Health (2020b). iCite. Available online at: https://icite.od.nih.gov (accessed January 29, 2020).

[B48] National Library of Medicine (2019). Funding Support (Grant) Information in MEDLINE/PubMed. Available online at: https://www.nlm.nih.gov/bsd/funding_support.html (accessed February 6, 2020).

[B49] National Research Council (2014a). Science of Science and Innovation Policy: Principal Investigators' Conference Summary. Washington, DC: The National Academies Press.

[B50] National Research Council (2014b). Capturing Change in Science, Technology, and Innovation: Improving Indicators to Inform Policy. Washington, DC: The National Academies Press.

[B51] National Science Foundation (2007). Science of Science Innovation and Policy FY 2007 Program Solicitation NSF 07-547. Available online at: https://www.nsf.gov/pubs/2007/nsf07547/nsf07547.htm (accessed January 22, 2020).

[B52] National Science Foundation (2019). Dear Colleague Letter: 2019 Social, Behavioral, and Economic (SBE) Repositioning. Available online at: https://www.nsf.gov/pubs/2019/nsf19089/nsf19089.jsp (accessed April 9, 2020).

[B53] Oancea A. (2013). Buzzwords and Values: The prominence of “impact” in UK research policy and governance. Res. Trends 33, 6–9. Available online at: https://www.researchtrends.com/issue-33-june-2013/buzzwords-and-values (accessed July 2, 2020).

[B54] Office of Science and Technology Policy (2006). The Science of Science Policy: A Federal Research Roadmap. Washington, DC: National Science and Technology Council.

[B55] Onken J. (2016). Improving the research portfolio data infrastructure at NIH, in Presentation, Modeling Science, Technology, and Innovation (Washington, DC). Available online at: https://modsti.cns.iu.edu/wp-content/uploads/2016/05/Onken_DAI.pdf (accessed July 8, 2020).

[B56] Pavlopoulos G. A. Paez-Espino D. Kyrpides N. C. Iliopoulos I. (2017). Empirical comparison of visualization tools for larger-scale network analysis. Adv. Bioinform. 2017:1278932. 10.1155/2017/1278932 28804499 PMC5540468

[B57] Questex (2020). FiercePharma: The Top 20 Drugs by 2018 U.S. Sales. Available online at: https://www.fiercepharma.com/special-report/top-20-drugs-by-2018-u-s-sales (accessed February 10, 2020).

[B58] Raftery J. Hanney S. Greenhalgh T. Glover M. Blatch-Jones A. (2016). Models and applications for measuring the impact of health research: update of a systematic review for the health technology assessment programme. Health Technol. Assess. 20, 1–254. 10.3310/hta20760 PMC5086596 27767013

[B59] RAND Europe. (2008). Medical Research: What's it Worth? Estimating the Economic Benefits From Medical Research in the UK. London: UK Evaluation Forum.

[B60] Research Excellence Framework (2015). REF Manager's Report. Available online at: https://www.ref.ac.uk/2014/media/ref/content/pub/REF_managers_report.pdf (accessed July 2, 2020).

[B61] Rigby J. (2011). Systematic grant and funding body acknowledgment data for publications: new dimensions and new controversies for research policy and evaluation. Res. Eval. 20, 365–375. 10.3152/095820211X13164389670392

[B62] Ruegg R. Jordan G. (2007). Overview of Evaluation Methods for R&D Programs: A Directory of Evaluation Methods Relevant to Technology Development Programs. Report prepared for U.S. Department of Energy Office of Energy Efficiency and Renewable Energy. Available online at: https://www1.eere.energy.gov/analysis/pdfs/evaluation_methods_r_and_d.pdf (accessed January 27, 2020).

[B63] Sherwin C. W. Isenson R. S. (1967). Project hindsight: defense department study of the utility of research. Science 156, 1571–1577. 10.1126/science.156.3782.1571 6025113

[B64] Skelton V. (2019). International consortium The Research on Research Institute launched. Information Today Europe. Available online at: https://www.infotoday.eu/Articles/News/Featured-News/International-consortium-The-Research-on-Research-Institute-launched-134441.aspx (accessed October 2, 2019).

[B65] Smith C. I. E. Zain R. (2019). Therapeutic oligonucleotides: state of the art. Annu. Rev. Pharmacol. Toxicol. 59, 605–630. 10.1146/annurev-pharmtox-010818-021050 30285540

[B66] Smith R. (1987). Comroe and dripps revisited. Br. Med. J. 295, 1404–1407. 10.1136/bmj.295.6610.1404 3690250 PMC1248552

[B67] U.S. Food Drug Administration (2020). Approved Drug Products with Therapeutic Equivalence Evaluations (Orange Book). Available online at: https://www.fda.gov/drugs/drug-approvals-and-databases/approved-drug-products-therapeutic-equivalence-evaluations-orange-book (accessed March 15, 2019).

[B68] UK Research Innovation (2020). The Gateway to Research: UKRI Portal Onto Publically Funded Research. Available online at: https://gtr.ukri.org/ (accessed July 9, 2020).

[B69] van Raan A. F. J. (2017). Patent citations analysis and its value in research evaluation: a review and a new approach to map technology-relevant research. J. Data Inf. Sci. 2, 13–50. 10.1515/jdis-2017-0002

[B70] Waldman L. Lariviere V. (2020). Special issue on bibliographic data sources. Quant. Sci. Stud. 1, 360–362. 10.1162/qss_e_00026

[B71] Williams R. S. Lotia S. Holloway A. K. Pico A. R. (2015). From scientific discovery to cures: bright stars within a galaxy. Cell 163, 21–23. 10.1016/j.cell.2015.09.007 26406364

[B72] Wooding S. Hanney S. Pollitt A. Buxton M. Grant J. (2011). Project Retrosight: Understanding the Returns From Cardiovascular and Stroke Research: The Policy Report. Santa Monica, CA: RAND Corporation. Available online at: https://www.rand.org/pubs/monographs/MG1079.html (accessed February 6, 2020). 10.7249/rhq-01-01-16 PMC4945223 28083172

[B73] Zuckerman B. L. Hautala J. A. Nek R. (2015). Technology Development at the National Institutes of Health (NIH): Summary Report (IDA Document D-5712). Washington DC: IDA Science & Technology Policy Institute.

